# P-Selectin Glycoprotein Ligand (PSGL)-1 Expression on CD4^+^ T Cells in Patients with HIV: Insights from an Observational Study

**DOI:** 10.3390/pathogens14121232

**Published:** 2025-12-03

**Authors:** Silvere D. Zaongo, Yan Wan, Lu Zhang, Shengquan Tang, Vijay Harypursat, Yaokai Chen

**Affiliations:** 1 Clinical Research Center, Chongqing Public Health Medical Center, 09 Baoyu Road, Geleshan Town, Shapingba District, Chongqing 400036, China; zsildieu@yahoo.fr (S.D.Z.); wany1005@163.com (Y.W.); 15023234577@163.com (L.Z.); shengquan_tang@outlook.com (S.T.); vijayharypursat@gmail.com (V.H.); 2 Department of Infectious Diseases, Chongqing Public Health Medical Center, 09 Baoyu Road, Geleshan Town, Shapingba District, Chongqing 400036, China

**Keywords:** HIV, PSGL-1, immunopathogenesis, CD4^+^ T-cell, ART-treated, ART-naïve

## Abstract

In the context of HIV infection, PSGL-1 expression and impact on CD4^+^ T cells remain largely unexplored. Thus, to address these critical gaps in knowledge, blood was sampled from HIV-negative controls and both ART-treated and ART-naïve individuals and stratified according to CD4^+^ T cell counts. PSGL-1 expression on CD4^+^ T cells and CD4^+^ T cell–platelet aggregates, along with PD-1, Bcl-2, and Caspase-3, were assessed with flow cytometry. Levels of IL-12, IFN-γ, LPS, and β-glucan were determined with ELISA. Spearman’s correlation test was used to determine the correlation between PSGL-1 expression on CD4^+^ T cell counts and markers of inflammation/translocation. PSGL-1 was significantly overexpressed in ART-treated individuals (*p* < 0.0001) and exhibited markedly lower expression in ART-naïve individuals (*p* ≤ 0.01), consistent with a suppressive influence of HIV VL prior to ART initiation and a proinflammatory environment promoting PSGL-1 overexpression during ART. In both groups, individuals with CD4^+^ T cells < 200 cells/µL exhibited elevated levels of PSGL-1 (*p* < 0.05) and increased CD4^+^ T cell–platelet aggregates (*p* < 0.05). In ART-treated individuals only, PSGL-1 expression positively correlated with IFN-γ (r = 0.318, *p* = 0.021), IL-12 (r = 0.498, *p* < 0.001), LPS (r = 0.382, *p* = 0.005), and β-glucan (r = 0.318, *p* = 0.021), reinforcing the link between inflammatory activation and PSGL-1 overexpression. In this group, those with CD4^+^ T cells < 200 cells/µL had higher Caspase-3 and PD-1 (*p* < 0.0001) and lower Bcl-2 (*p* ≤ 0.01). No significant differences in these markers were found across CD4^+^ strata in ART-naïve individuals. PSGL-1 expression is influenced by ART and immune status. PSGL-1 signaling drives CD4^+^ T cell phenotypic changes.

## 1. Introduction

P-selectin glycoprotein ligand 1 (PSGL-1) is a proinflammatory cell surface molecule that is expressed on leukocytes [1]. PSGL-1 expression is triggered by inflammation [2,3,4,5], and its role as a transmembrane receptor is to recruit immune cells from within the bloodstream and localize them to inflamed organs and tissues [6,7,8,9,10] via the attachment of PSGL-1 to different selectin molecules located on specific cells (P-selectin on platelets, E-selectin on endothelial cells, and L-selectin on leukocytes) [11].

In the context of HIV infection, PSGL-1 exhibits multifaceted roles, serving both as an HIV restriction factor and as a potential mediator of viral transmission. As an HIV restriction factor, PSGL-1 on infected cells (i) induces the production of HIV virions lacking the critical gp120 and gp41 envelope glycoproteins, thus inhibiting virion infectivity [12,13], and (ii) inhibits the HIV reverse transcription process by capturing F-actin, a component essential for successful cDNA synthesis [14]. These functions highlight the contribution of PSGL-1 to limiting viral replication and spread. Conversely, PSGL-1 may also facilitate the transmission of HIV to uninfected cells. Notably, PSGL-1 has the capacity to incorporate into the enveloping membrane of new virions even at a low level of PSGL-1 expression on cells [14]. Interestingly, virions produced from infected cells expressing low levels of PSGL-1 retain the ability to infect uninfected cells. This is facilitated by the incorporated PSGL-1, which mediates binding to selectins on the surface of target cells, promoting viral entry [15]. This dual functionality underscores the complex and versatile role of PSGL-1 during HIV infection, acting as both an obstacle to, and a potential promotor of, viral propagation.

Given that CD4^+^ T cells are the primary targets of HIV infection, investigating the levels of expression of PSGL-1 on these cells is of significant interest. However, the expression of PSGL-1 in people living with HIV (PLWH) has been explored in only a limited number of published studies to date. Connor et al. [16] have observed that compared to healthy individuals, ART-treated individuals with undetectable viral loads (VLs) display higher levels of PSGL-1 on monocytes. In contrast, Liang et al. [17] have observed that compared to healthy controls, the expression of PSGL-1 on monocytes is significantly decreased in untreated PLWH. These studies suggest that PSGL-1 expression on monocytes increases in ART-treated individuals and decreases in ART-naïve individuals, implying differential regulation of PSGL-1 expression in the contexts of treated and untreated HIV infection. Nevertheless, while these studies provide valuable insights into PSGL-1 dynamics, they do not specifically address the expression of PSGL-1 on CD4^+^ T cells. Notably, findings observed in monocytes cannot be directly extrapolated to CD4^+^ T cells, as results in one cell type may not necessarily apply to another. This knowledge gap is particularly consequential given the inconsistent immunological responses observed among ART-treated individuals. Indeed, despite ART successfully clearing HIV VL from the bloodstream, a significant subset of ART-treated individuals (15–40%), referred to as immunological non-responders (INRs), fail to achieve robust CD4^+^ T cell recovery [18]. Thus, it is also necessary and important to observe PSGL-1 expression in these sub-categories of ART-treated individuals, as they may exhibit differing patterns of PSGL-1 expression. Further, the associations between PSGL-1 expression and plasma levels of proinflammatory markers are also worthy of exploration, given that HIV infection is a chronic infection known to be associated with persistent and enduring inflammation/microbial translocation. Furthermore, it has been shown that PSGL-1 sustains the expression of inhibitory receptors such as PD-1, thereby promoting T cell exhaustion during chronic viral infections [19]. Thus, a thorough understanding of the variations in PSGL-1 expression and their implications for CD4^+^ T cell exhaustion, survival, and death is crucial. Knowledge gleaned from the study of these information gaps may inform the design and development of targeted therapies aimed at modulating PSGL-1 activity to enhance immunological function, control HIV replication, and perhaps encourage immune recovery in INRs.

In this study, we use a cross-sectional approach to explore PSGL-1 expression on CD4^+^ T cells across different categories of PLWH (both ART-treated and ART-naïve). We also assess the proportions of CD4^+^ T cell–platelet aggregates and measure levels of markers associated with inflammation, cellular exhaustion, survival, and cell death and analyze their relationships with PSGL-1 expression.

## 2. Materials and Methods

### 2.1. Study Population, Inclusion, and Exclusion Criteria

Participants were enrolled at Chongqing Public Health Medical Center (which is the designated HIV testing and treatment center for the city and region) between January 2024 to September 2024. Patients with HIV were invited to participate in the study if they had been on prescribed ART for at least two continuous years and displayed undetectable VLs (<50 RNA copies/mL) or if they were ART-naïve with a VL of >1000 copies/mL at the time of their enrolment. These strict inclusion criteria were chosen to clearly distinguish between individuals with controlled viral replication (ART-treated) and those with ongoing viral replication (ART-naïve). We also recruited HIV-negative adults as controls. HIV-negative controls were selected from a pool of individuals who tested negative for HIV and who voluntarily agreed to participate in the study. They were matched based on age and sex to minimize potential confounding effects. Participants were excluded if they (1) had a chronic disease (cancer, HBV, diabetes, kidney disease, chronic lung disease, and cardiovascular diseases, amongst others) at the time of enrolment, (2) were found to have organ failure or were in a decompensated state, (3) were pregnant or breastfeeding, (4) were below 18 years of age, or (5) refused to sign informed consent. Overall, 130 participants (63 ART-treated, 39 ART-naïve, and 28 HIV-negative) were screened, as they met these criteria.

### 2.2. Ethical Considerations

This study was reviewed and approved by the ethics committee of Chongqing Public Health Medical Center (approval number: 2023-049-01-KY; Chongqing, China). Each participant was required to provide written informed consent at study enrolment. Notably, this study followed the recommendations for human participants and the guidelines provided by the Declaration of Helsinki. Thus, confidentiality and privacy were rigorously upheld in strict alignment with established clinical protocols. Participants were assured of the right to withdraw from the study at any point without any impact on the quality or continuity of their medical care. Throughout all procedures, study supervisors diligently ensured adherence to ethical standards, consistently protecting the rights, autonomy, and dignity of all participants. Finally, all experiments presented in this study were performed in accordance with relevant guidelines and regulations.

### 2.3. Clinical Samples and Isolation of PBMCs

Ten milliliters (mL) of blood was collected from each participant in EDTA tubes and was immediately transferred to the laboratory, where peripheral blood mononuclear cells (PBMCs) were isolated by Ficoll density gradient centrifugation. A portion of the PBMC sample was stored at −80 °C for subsequent analysis. Notably, after consenting to provide blood samples, participants in the ART-naïve group were educated about ART and its benefits and were encouraged to seek treatment.

### 2.4. HIV-1 Viral Load and CD4^+^ and CD8^+^ T Cell Counts

HIV-1 VL was determined using the COBAS AmpliPrep/COBAS Taqman HIV-1 Test (V2.0 platform), using 1.2 mL of freshly collected plasma. CD4^+^ and CD8^+^ T cell counts were determined following the procedure published by Dillon et al. [20], using flow cytometry on freshly collected whole blood. CD4^+^ T cells and CD8^+^ T cells were identified as CD3^+^CD4^+^ and CD3^+^CD8^+^ cells, respectively.

### 2.5. PSGL-1 Profiling, CD4^+^ T Cell–Platelet Aggregates, and CD4^+^ T Cell Signatures

With respect to PSGL-1 profiling, PBMCs allocated to flow cytometry assessment were immediately labeled with fluorescently conjugated antibodies or reagents such as anti-CD3 (Biolegend, San Diego, CA, USA), anti-CD4 (Biolegend), and anti-PSGL-1 (Biolegend). CD4^+^ T cell–platelet aggregates were assessed using anti-CD42a (Thermo Fisher, Waltham, MA, USA). Furthermore, a range of antibodies, including anti-PD-1 (BioLegend), anti-BCL-2 (BioLegend), and anti-Caspase-3 (BD Biosciences, Milpitas, CA, USA) were used to assess markers of exhaustion, survival, and apoptotic signatures, respectively, in CD4^+^ T cells either independently or in conjunction with platelets, as CD4^+^ T cell–platelet aggregates. The aforementioned cell specific markers were detected during flow cytometry, according to the protocols specified by the manufacturer. Data were acquired using a BD Canto II flow cytometer (BD Biosciences) and analyzed using FlowJo 10.9.0 software (Tree Star, Woodburn, OR, USA).

### 2.6. Detection of Specific Markers of Inflammation Using ELISA

ELISA kits for IL-12 (Jiangsu Meibiao Biotechnology (Yancheng, China), MB-0063B) and IFN-γ (Jiangsu Meibiao Biotechnology, MB-0033A) detection were used to evaluate the levels of these proinflammatory cytokines, which have been previously described as inducers of PSGL-1 expression [12,21]. Additionally, the levels of lipopolysaccharides (LPS) (Jiangsu Meibiao Biotechnology, MB-00349A) and beta (β)-glucan (Jiangsu Meibiao Biotechnology, MB-00357A) were assessed. All experiments were conducted in strict accordance with the manufacturer’s instructions. The SpectraMax ABS Plus microplate reader (monitored by SoftMax Pro7.1 software) was utilized to read absorbance and to quantify the expression of each cytokine.

### 2.7. Collection of Clinical Data

At the time of the laboratory analyses, supplementary data for each patient were retrieved from the hospital records database. This included details on age, sex, ART regimen and duration, and CD4^+^ T cell counts at enrolment.

### 2.8. Data Analysis

The data were collected and analyzed in GraphPad Prism software (version 10.2.2, Boston, MA, USA). The distribution of continuous variables was assessed using the Shapiro–Wilk test. Variables following a normal distribution are presented as means ± standard deviations (SDs), whereas non-normally distributed variables are expressed as medians with interquartile ranges (IQRs). Non-parametric tests were used to analyze non-normally distributed variables. The Mann–Whitney U test was used to compare data from two unpaired groups. For comparisons involving three groups, the Kruskal–Wallis test was performed. Comparisons were conducted between HIV-negative individuals and those who were either ART-treated or ART-naïve, as well as between subgroups within the ART-treated or ART-naïve groups based on CD4^+^ T cell counts (<200 cells/µL and ≥200 cells/µL). Spearman’s correlation was used to determine the correlation between PSGL-1 and plasma markers of inflammation/microbial translocation in individuals who were either ART-treated or ART-naïve. The statistical significance level for all tests was defined as a 2-tailed *p*-value of <0.05.

## 3. Results

### 3.1. Clinical Characteristics of Enrolled Participants

Altogether, 117 participants were enrolled, including 26 HIV-negative individuals, 52 ART-treated individuals (26 with CD4^+^ T cell counts < 200 cells/µL and 26 with CD4^+^ T cell counts ≥ 200 cells/µL), and 39 ART-naïve individuals (15 with CD4^+^ T cell counts < 200 cells/µL and 24 with CD4^+^ T cell counts ≥ 200 cells/µL). Across all groups, women represented approximately 20% of the study population. Detailed demographic and clinical characteristics are presented in Table 1.

### 3.2. PSGL-1 Expression on CD4^+^ T Cells

**Figure 1:**
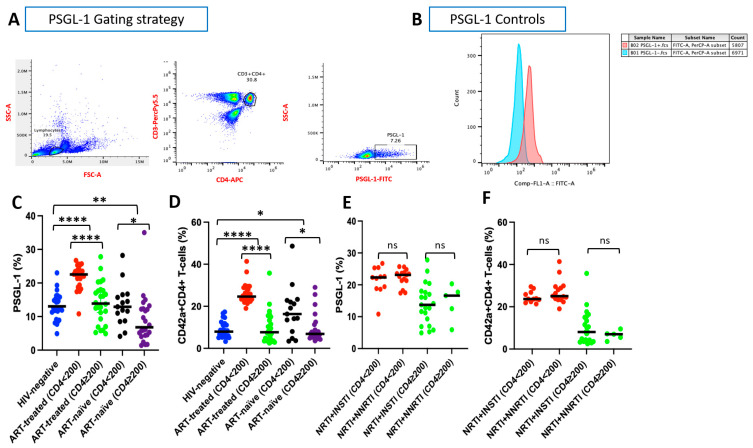
PSGL-1 expression and CD4^+^ T cell–platelet aggregates. The panel presents the proportions of PSGL-1 expression on CD4^+^ T cells (**A**–**C**), the proportions of CD4^+^ T cell–platelet aggregates (**D**), and the effects of ART on both PSGL-1 expression (**E**) and CD4^+^ T cell–platelet aggregates (**F**). * *p* < 0.05; ** *p* ≤ 0.01; **** *p* < 0.0001; ns: not statistically significant. In D, *p* = 0.740 and *p* = 0.613 between HIV-negative and ART-treated individuals (CD4^+^ ≥ 200 cells/µL) and between HIV-negative and ART-naïve individuals (CD4^+^ ≥ 200 cells/µL), respectively. Blue dots: data from HIV-negative; red dots: data from ART-treated individuals with CD4^+^ < 200 cells/µL; green dots: data from ART-treated individuals with CD4^+^ ≥ 200 cells/µL; black dots: data from ART-naïve individuals with CD4^+^ < 200 cells/µL; purple dots: data from ART-treated individuals with CD4^+^ ≥ 200 cells/µL. Black lines represent the median values of PSGL-1 or CD4⁺ T cell–platelet aggregates for each category of individuals.

We evaluated PSGL-1 expression by analysis of the proportion of CD4^+^ T cells expressing PSGL-1 relative to the total CD4^+^ T cell population (PSGL-1^+^ CD4^+^ T cells/total CD4^+^ T cells; Figure 1A–C). Among ART-treated individuals, those with CD4^+^ T cell counts < 200 cells/µL exhibited significantly higher proportions of PSGL-1 expression on their CD4^+^ T cells compared to individuals with CD4^+^ T cell counts ≥ 200 cells/µL. Notably, the proportions of PSGL-1 expression in ART-treated individuals with CD4^+^ T cells ≥ 200 cells/µL were comparable to those observed in HIV-negative controls (*p* = 0.46).

Similarly, among ART-naïve individuals, the proportions of PSGL-1 expression were significantly higher in those with CD4^+^ T cell counts < 200 cells/µL than in individuals with CD4^+^ T cell counts ≥ 200 cells/µL. Interestingly, ART-naïve individuals with CD4^+^ T cell counts < 200 cells/µL displayed proportions of PSGL-1 expression similar to those of HIV-negative controls (*p* = 0.97).

**Figure 2:**
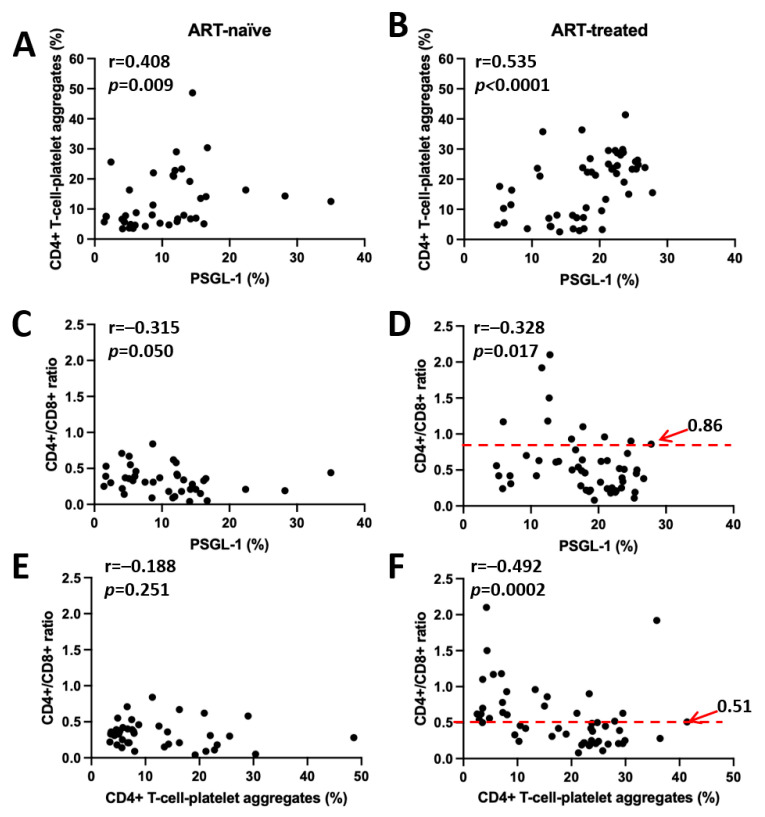
Correlations among PSGL-1 expression, CD4^+^ T cell–platelet aggregates, and CD4^+^/CD8^+^ ratio in ART-treated and ART-naïve individuals. Panels on the left (**A**,**C**,**E**) present correlations in ART-naïve individuals, while panels on the right (**B**,**D**,**F**) show correlations in ART-treated individuals. (**A**,**B**): correlations between PSGL-1 expression and CD4^+^ T cell–platelet aggregates. (**C**,**D**): correlations between PSGL-1 expression and the CD4^+^/CD8^+^ ratio. (**E**,**F**): correlations between CD4^+^ T cell–platelet aggregates and the CD4^+^/CD8^+^ ratio.

Furthermore, we observed that specific ART regimens, either NRTI + INSTI or NRTI + NNRTI regimens, did not affect PSGL-1 expression on CD4^+^ T cells in ART-treated individuals. As such, the proportions of PSGL-1 expression remained consistent between individuals on ART with CD4^+^ T cell counts < 200 cells/µL and those on ART with counts ≥ 200 cells/µL (Figure 2A).

### 3.3. CD4^+^ T Cell–Platelet Aggregates

We evaluated the proportion of CD4^+^ T cell–platelet aggregates (CD42a^+^ CD4^+^ T cells/total CD4^+^ T cells) to determine their relationship with the proportion of PSGL-1 expression on CD4^+^ T cells, as CD4^+^ T cell and platelet aggregates are mediated through PSGL-1 and P-selectin attachment. HIV-negative controls exhibited significantly lower proportions of CD4^+^ T cell–platelet aggregates compared to PLWH (*p* < 0.0001 versus ART-treated individuals and *p* = 0.026 versus ART-naïve individuals; Figure 1D).

Among ART-treated individuals, those with CD4^+^ T cell counts < 200 cells/µL showed significantly higher proportions of CD4^+^ T cell–platelet aggregates compared to those with CD4^+^ T cell counts ≥ 200 cells/µL (24.65% versus 7.65%; *p* < 0.001). Similarly, in ART-naïve individuals, participants with CD4^+^ T cell counts < 200 cells/µL displayed elevated proportions of CD4^+^ T cell–platelet aggregates compared to their counterparts with CD4^+^ T cell counts ≥ 200 cells/µL (16.30% versus 6.86%; *p* = 0.022).

Additionally, different ART regimens (NRTI + INSTI or NRTI + NNRTI in this instance) were observed to not significantly alter the profiles of CD4^+^ T cell–platelet aggregates among ART-treated individuals (Figure 2B).

Our observations revealed a distinct pattern between the proportion of CD4^+^ T cell–platelet aggregates and the proportion of PSGL-1 expression on CD4^+^ T cells. Specifically, both ART-treated and ART-naïve individuals with CD4^+^ T cells < 200 cells/µL exhibited higher proportions of PSGL-1 expression on CD4^+^ T cells and were associated with higher proportions of CD4^+^ T cell–platelet aggregates (Figure 1C,D) in both groups.

### 3.4. Correlation Between PSGL-1 Expression, CD4^+^ T Cell–Platelet Aggregates, and CD4^+^/CD8^+^ Ratio

To further comprehend the associations between PSGL-1 expression and the proportions of CD4^+^ T cell–platelet aggregates, we tested their correlations. Our results (Figure 2A,B) indicate that PSGL-1 expression positively correlates with the proportion of CD4^+^ T cell–platelet aggregates in both ART-treated (r = 0.535, *p* < 0.0001) and ART-naïve individuals (r = 0.408, *p* = 0.009).

In addition, the associations between PSGL-1 expression and CD4^+^/CD8^+^ ratio were also explored (Figure 2C,D). Our results showed a significant negative association between the CD4^+^/CD8^+^ ratio and PSGL-1 expression in ART-treated individuals (r = −0.328, *p* = 0.017). The CD4^+^/CD8^+^ ratio cut-off associated with higher PSGL-1 expression was 0.86. No statistically significant correlation was observed between the CD4^+^/CD8^+^ ratio and PSGL-1 expression in ART-naïve individuals (r = −0.315, *p* = 0.050).

Analysis of the relationship between the CD4^+^/CD8^+^ ratio and CD4^+^ T cell–platelet aggregates in both ART-treated and ART-naïve individuals (Figure 2E,F) showed that a significant negative correlation was present only in the ART-treated group (r = −0.492, *p* = 0.0002), whereas the association in ART-naïve individuals did not reach statistical significance (r = −0.188, *p* = 0.251). In ART-treated individuals, the CD4^+^/CD8^+^ ratio cut-off associated with a higher proportion of CD4^+^ T cell–platelet aggregates was 0.51.

### 3.5. Correlation Between PSGL-1 Expression and Plasma Markers of Inflammation/Microbial Translocation

**Figure 3:**
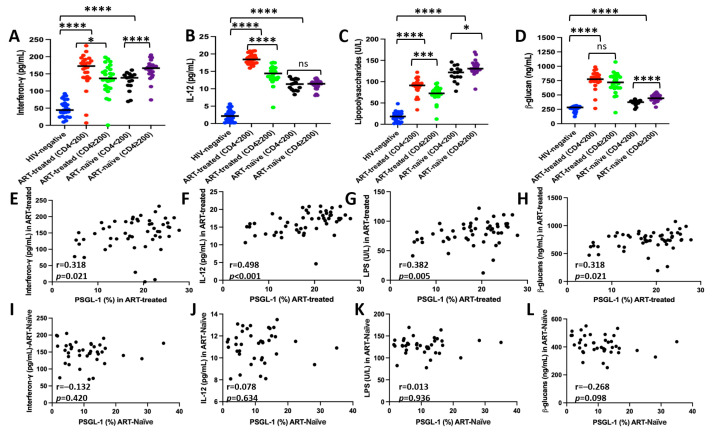
Levels of plasma markers in each group of people living with HIV (**A**–**D**) and their correlation with the proportions of PSGL-1 expression on CD4+ T cells (**E**–**L**). LPS: lipopolysaccharides; ns: not statistically significant. * *p* < 0.05; *** *p* ≤ 0.001; **** *p* < 0.0001. Blue dots: data from HIV-negative; red dots: data from ART-treated individuals with CD4^+^ < 200 cells/µL; green dots: data from ART-treated individuals with CD4^+^ ≥ 200 cells/µL; black dots: data from ART-naïve individuals with CD4^+^ < 200 cells/µL; purple dots: data from ART-treated individuals with CD4^+^ ≥ 200 cells/µL. Black lines in subfigures A–D represent the median plasma marker values for each category of individuals.

We measured the plasma levels of specific markers associated with inflammation and microbial translocation in both ART-treated and ART-naïve individuals. Then, we analyzed the associations between the proportions of PSGL-1 expression on CD4^+^ T cells and the plasma markers. As shown in Figure 3A–D, our observations revealed that PLWH, regardless of ART status, exhibited significantly elevated levels of IFN-γ (*p* < 0.0001), IL-12 (*p* < 0.0001), LPS (*p* < 0.0001), and β-glucan (*p* < 0.0001) compared to HIV-negative controls.

Among ART-treated individuals, those with CD4^+^ T cell counts < 200 cells/µL demonstrated significantly higher levels of IFN-γ (172.6 pg/mL versus 136.8 pg/mL, *p* = 0.012), IL-12 (18.42 pg/mL versus 14.40 pg/mL, *p* < 0.0001), and LPS (91.28 U/L versus 72.54 U/L, *p* = 0.0001) compared to individuals with CD4+ T cell counts ≥ 200 cells/µL. However, β-glucan levels were observed to not differ significantly between these two groups (719 ng/mL versus 774.4 ng/mL, *p* = 0.157).

In ART-naïve individuals, those with CD4^+^ T cell counts ≥ 200 cells/µL displayed significantly higher levels of IFN-γ (166.5 pg/mL versus 138.4 pg/mL, *p* < 0.0001), LPS (130.6 U/L versus 121.8 U/L, *p* = 0.013), and β-glucan (442.8 ng/mL versus 375 ng/mL, *p* < 0.0001) compared to individuals with CD4^+^ T cell counts < 200 cells/µL. However, IL-12 levels were identical between these two groups (11.42 pg/mL versus 11.34 pg/mL, *p* = 0.98).

Correlation analysis in ART-treated individuals revealed significant positive associations between the proportions of PSGL-1 expression and plasma levels of IFN-γ (r = 0.318, *p* = 0.021), IL-12 (r = 0.498, *p* < 0.001), LPS (r = 0.382, *p* = 0.005), and β-glucan (r = 0.318, *p* = 0.021) (Figure 3E–H). In contrast, no significant correlations were observed between the proportions of PSGL-1 expression and the aforementioned markers in ART-naïve participants (Figure 3I–L).

### 3.6. Impact of PSGL-1 on Markers of Death, Survival, and Exhaustion of CD4^+^ T Cells

**Figure 4:**
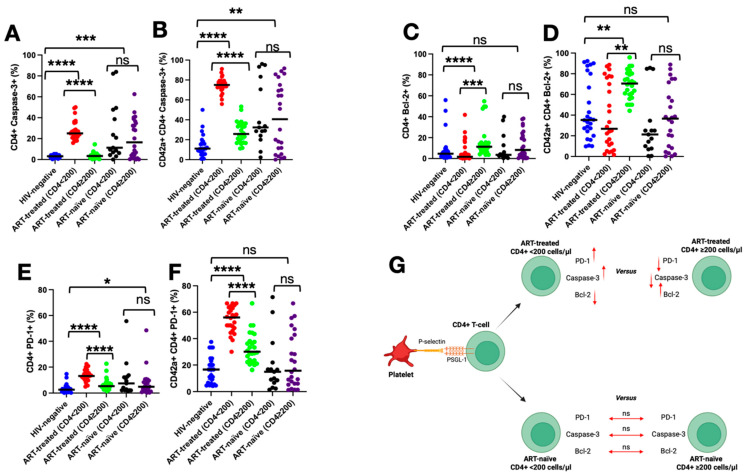
Proportions of CD4^+^ T cells expressing Caspase-3 (**A**,**B**), Bcl-2 (**C**,**D**), and PD-1 (**E**,**F**). (**G**) illustrates the phenotypic profiles of CD4^+^ T cells when engaged in aggregates with platelets. ns: not statistically significant. * *p* < 0.05; ** *p* ≤ 0.01; *** *p* ≤ 0.001; **** *p* < 0.0001. Blue dots: data from HIV-negative; red dots: data from ART-treated individuals with CD4^+^ < 200 cells/µL; green dots: data from ART-treated individuals with CD4^+^ ≥ 200 cells/µL; black dots: data from ART-naïve individuals with CD4^+^ < 200 cells/µL; purple dots: data from ART-treated individuals with CD4^+^ ≥ 200 cells/µL. Black lines represent the median values of caspase-3, Bcl-2, or PD-1 for each category of individuals. In subfigure G, the black arrow indicates the cases, and the red arrows represent the levels of marker expression. Red up arrow: increased expression; Red down arrow: reduced expression; double-headed horizontal arrow: no statistically significant change in expression.

We analyzed the impact of PSGL-1 expression on CD4^+^ T cell phenotypes by assessing the proportions of CD4^+^ T cells (both individually and in conjunction with platelets as CD4^+^ T cell–platelet aggregates) expressing Caspase-3, Bcl-2, and PD-1. Our observations revealed that, compared to CD4^+^ T cells alone (Figure 4A,C,E), CD4^+^ T cells forming aggregates with platelets (Figure 4B,D,F) exhibited significantly higher proportions of Caspase-3 (*p* < 0.0001), Bcl-2 (*p* = 0.0007), and PD-1 (*p* < 0.0001) across all categories of PLWH and HIV-negative controls. Our observations suggest a critical role for PSGL-1 signaling in the initiation and propagation of these phenotypic alterations in CD4^+^ T cells.

Among ART-treated individuals, our observations revealed that PSGL-1 may differentially modulate these phenotypes based on the immune status of an individual. Phenotypic analysis of CD4^+^ T cells alone revealed that participants with CD4^+^ T cell counts < 200 cells/µL showed markedly elevated proportions of Caspase-3 (25.05% compared to 3.335% in participants with CD4^+^ T cell counts ≥ 200 cells/µL, *p* < 0.0001) and PD-1 (13.25% compared to 5.375% in participants with CD4^+^ T cell counts ≥ 200 cells/µL, *p* < 0.0001). This trend was amplified in CD4^+^ T cells forming aggregates with platelets, where individuals with CD4^+^ T cell counts < 200 cells/µL showed even higher proportions of Caspase-3 (75% compared to 25.75% in participants with CD4^+^ T cell counts ≥ 200 cells/µL, *p* = 0.0001) and PD-1 (56.05% compared to 30.10%, in participants with CD4^+^ T cell counts ≥ 200 cells/µL, *p* = 0.0001) (Figure 4A,B,E–G). Conversely, participants with CD4^+^ T cell counts ≥ 200 cells/µL exhibited significantly higher proportions of Bcl-2, ranging from 11.3% in CD4^+^ T cells alone (compared to 1.715% in participants with CD4^+^ T cell counts < 200 cells/µL, *p* = 0.0003) to 70.50% in CD4^+^ T cells forming aggregates with platelets (compared to 26.80% in participants with CD4^+^ T cell counts < 200 cells/µL, *p* = 0.001) (Figure 4C,D,G).

In the ART-naïve group, the impact of PSGL-1 appeared negligeable when assessed in the context of an individual’s immune status. When analyzing the phenotypes of CD4^+^ T cells alone, the proportions of cells expressing Caspase-3 (*p* = 0.506), Bcl-2 (*p* = 0.306), and PD-1 (*p* = 0.313) were comparable between individuals with CD4^+^ T cell counts < 200 cells/µL and those with CD4^+^ T cell counts ≥ 200 cells/µL. Similarly, in CD4^+^ T cells forming aggregates with platelets, the proportions expressing Caspase-3 (*p* = 0.403), Bcl-2 (*p* = 0.419), and PD-1 (*p* = 0.759) were also consistent across these two CD4^+^ T cell count groups (Figure 4A–G).

## 4. Discussion

Herein, we have assessed PSGL-1 expression on CD4^+^ T cells, CD4^+^ T cell–platelet aggregates, and plasma markers of inflammation and microbial translocation in ART-treated and ART-naïve individuals. Additionally, we have examined the relationships between PSGL-1 expression and these markers, as well as the impact of PSGL-1 expression in distinct CD4^+^ T cell phenotypic strata.

We have provided insights into PSGL-1 expression on CD4^+^ T cells (Figure 1C) in this study. Notably, when compared to HIV-negative controls, PSGL-1 expression across dissimilar categories of PLWH reveals distinct patterns. In ART-treated individuals, PSGL-1 is significantly overexpressed (*p* < 0.0001). This is likely due to the persistent chronic inflammation observed in ART-treated PLWH, as has been reported in previous publications [22,23]. Given that PSGL-1 expression is triggered by inflammation [2,3,4,5], we believe that the chronic inflammation noted in ART-treated individuals may act as a driver that sustains their elevated PSGL-1 levels. Conversely, in ART-naïve individuals, PSGL-1 expression on CD4^+^ T cells does not exceed levels observed in HIV-negative controls. In fact, PSGL-1 expression is suppressed in this group (*p* ≤ 0.01). This suppression may be attributable to high HIV VLs (Table 1), as prolific HIV replication encourages Vpu and Nef [12,13] to downregulate PSGL-1 expression. Consequently, ART-naïve individuals are likely to exhibit a lower degree of PSGL-1 expression. Collectively, these observations suggest that PSGL-1 primarily serves as an HIV restriction factor counteracted by HIV VLs in ART-naïve individuals and predominantly exhibits proinflammatory activity in those undergoing ART. Importantly, our observations suggest that PSGL-1 expression may serve as a distinctive marker of immune status in both ART-treated and ART-naïve PLWH (Figure 1C). Nonetheless, given the novelty of our observations, further research is necessary to substantiate and validate their clinical significance. Additionally, we have demonstrated that HIV infection promotes the formation of CD4^+^ T cell–platelet aggregates (Figure 1D). Our observations show that in both ART-treated and ART-naïve contexts, individuals with elevated PSGL-1 expression on CD4^+^ T cells are more likely to exhibit higher levels of CD4^+^ T cell–platelet aggregates (Figure 1C,D). Among ART-treated individuals, CD4^+^ T cell–platelet aggregates are significantly higher compared to HIV-negative controls (*p * < 0.0001, Figure 1D), aligning with the outcomes of previous studies [24,25]. An analysis based on immune status further corroborates the results Dai et al. [24] who observed that ART-treated PLWH with poor immune recovery exhibit higher proportions of CD4^+^ T cell–platelet aggregates compared to immunological responders. Such a scenario contributes to further CD4^+^ T cells loss in INRs, and we believe that in the context of our study, the elevated expression of PSGL-1 and the higher proportions of CD4^+^ T cell–platelet aggregates potentially contribute to maintenance of low levels of CD4^+^ T cells in ART-treated individuals with CD4^+^ T cell counts < 200 cells/µL. For ART-naïve individuals, we observed higher levels of CD4^+^ T cell–platelet aggregates compared to HIV-negative controls (*p* < 0.05, Figure 1D), which is consistent with the observations of previous reports in ART-naïve populations [17,24]. This outcome is intriguing, as PSGL-1 expression is significantly suppressed in ART-naïve individuals, suggesting that the formation of CD4^+^ T cell–platelet aggregates, in the ART-naïve context, does not solely depend on the levels of PSGL-1 expressed on CD4^+^ T cells. However, at the immune status level, individuals with CD4^+^ T cell counts < 200 cells/µL were observed to have higher PSGL-1 expression and higher levels of CD4^+^ T cell–platelet aggregates. This observation may explain why ART naïve individuals with CD4^+^ T cell counts < 200 cells/µL also display reduced overall CD4^+^ T cell counts. Due to the novelty of our findings with respect to ART-naïve individuals, the absence of comparable data in the existing literature poses challenges for deeper interpretation of our data. Nevertheless, it is worth noting that our observation of elevated CD4^+^ T cell–platelet aggregates, associated with poor immune status (CD4^+^ T cell < 200 cells/μL) in both ART-treated and ART-naïve individuals, is clinically significant. Beyond reflecting severe CD4^+^ T cell depletion, these aggregates may also indicate an increased risk of cardiovascular events [26], particularly among ART-treated individuals who may live longer while experiencing persistent chronic inflammation. Accordingly, CD4^+^/CD8^+^ ratios below the identified cut-offs (Figure 2D,F; 0.86 and 0.51, respectively), which are associated with higher PSGL-1 expression and higher proportions of CD4^+^ T cell–platelet aggregates, may serve as useful indicators of these risks in ART-treated individuals.

In our study, we have demonstrated that in the context of HIV infection, PSGL-1 expression is variably associated with markers of inflammation and microbial translocation and is dependent on ART status (Figure 3). Notably, no discernible association with the aforementioned markers was observed in ART-naïve individuals. In contrast, in ART-treated individuals, at least 31% of the variation in PSGL-1 expression on CD4^+^ T cells may be attributable to plasma levels of IFN-γ, IL-12, β-glucan, and LPS (Figure 3E–H). These observations suggest that markers of inflammation and microbial translocation may modulate PSGL-1 expression in ART-treated individuals. The previously documented influence of IFN-γ and IL-12 on PSGL-1 expression [12,27] aligns with our observations. However, with respect to IL-12, our study reports (for the first time) the potential role that IL-12 may have in PSGL-1 expression in humans. Previous evidence regarding this [27] was derived from a murine Th1 cell model. Furthermore, this study provides novel evidence of an association between microbial translocation markers and PSGL-1 expression. Our team has previously hypothesized that microbial translocation might broadly influence PSGL-1 expression in PLWH [28]. The observations presented here refine this hypothesis, indicating that the impact of microbial translocation products on PSGL-1 expression is likely to be limited to ART-treated individuals with undetectable HIV VLs.

This study indicates that in the context of HIV infection, inflammation and microbial translocation induce PSGL-1 expression on CD4^+^ T cells, which in turn contributes to the formation of CD4^+^ T cell–platelet aggregates. Notably, the elevated proportions of these aggregates, observed by our team and others [17,24,25], suggest that PSGL-1 may play a role in modulating CD4^+^ T cell functions. Indeed, previous research has shown that PSGL-1 expression and its engagement with P-selectin or anti-PSGL-1 antibodies may inhibit the proliferation of human hematopoietic stem cells [29] and modulate T cell receptor (TCR) signaling [30]. For these reasons, PSGL-1 is regarded as an immune checkpoint [21], meaning that its downstream signaling modulates immune cell responses. Herein, we have investigated the potential roles of PSGL-1 in CD4^+^ T cell death, survival, and exhaustion (Figure 4A–F) across distinct categories of PLWH. Overall, PSGL-1 engagement with P-selectin promotes Caspase-3, Bcl-2, and PD-1 expression in CD4^+^ T cells. On the one hand, PSGL-1 enhances cell death and exhaustion in ART-treated individuals with CD4^+^ T cell counts < 200 cells/µL, and these results align with previous observations [24]. On the other hand, PSGL-1 promotes cell survival in ART-treated individuals having CD4^+^ T cell counts ≥ 200 cells/µL. This aspect of the role of PSGL-1 in cell survival differs from what has been reported previously. Indeed, Dai et al. [24] have observed that CD4^+^ T cells exhibit decreased Bcl-2 levels when PSGL-1 is engaged by P-selectin on platelets. This discrepancy between our results and those of Dai et al. is believed to arise from differences in experimental protocols. For instance, in our study, samples were processed immediately after collection, whereas the methods described by Dai et al. do not specify whether samples were stored or used immediately. Additionally, their stratification criteria for PLWH differ from those used in our investigation. It is also known that PSGL-1 may induce T cell death through caspase-independent pathways involving apoptotic mediators such as apoptosis-inducing factor (AIF) and cytochrome c [31]. While Bcl-2 inhibits apoptosis by preventing AIF release, this relationship is complex. AIF may still induce apoptosis even in the presence of Bcl-2, suggesting that Bcl-2 does not completely block the effects of AIF [32]. Our observations suggest that the downstream signaling of PSGL-1 operates via mechanisms that are not as yet fully understood or appreciated. In ART-naïve individuals, Caspase-3, Bcl-2, and PD-1 levels are comparable across CD4^+^ T cell strata. Similarly, CD4^+^ T cells interacting with platelets exhibit consistent marker profiles, regardless of CD4^+^ T cell counts. Compared against ART-treated individuals, high HIV VLs in ART-naïve individuals are likely to distort the effects of PSGL-1 on CD4^+^ T cells. Overall, our observations highlight the complex and context-specific roles of PSGL-1 in HIV infection and immune regulation.

We acknowledge that our study has several limitations. The study population has a significant bias toward male participants (~80%). Given that biological sex may influence immune responses and PSGL-1 expression, this gender bias may limit the generalizability of the findings to women living with HIV. A deeper analysis of CD4^+^ T cell engagement with platelets would have provided further details and understanding regarding the role of PSGL-1 in the context of HIV infection. Indeed, we looked only for the CD42a marker to study the interactions of CD4^+^ T cells with platelets, and we could not, with any degree of certainty, ascertain whether the cells studied where effectively individual cells and were not concurrently engaged in interactions with other cells via other receptors. Furthermore, the phenotypic profiling of CD4^+^ T cells would have been necessary for this to have been accurately studied, as it is known that during HIV infection, several subtypes of CD4^+^ T cells (naïve, effector, precursor, memory, and others) may be encountered. As such, it is likely that our observations may be dependent on the subtypes of cells involved. Perhaps, the application of single cell sequencing and tools for signaling pathway prediction could have been utilized. That said, it is worth emphasizing that investigating the proportions of CD4^+^ T cell–platelet aggregates is critical, as elevated PSGL-1 expression on CD4^+^ T cells does not necessarily indicate that all PSGL-1 molecules will interact with P-selectin. However, PSGL-1 must undergo glycosylation [specifically, the formation of sialyl Lewis x (sLex)-capped O-glycans] before it may bind to selectins, including P-selectin [9,33]. Therefore, profiling the expression of enzymes responsible for this glycosylation are likely to provide deeper insights into the fundamental mechanisms underlying the formation of CD4^+^ T cell–platelet aggregates during HIV infection. Finally, the relatively lower sample size of ART-naïve individuals enrolled in this study may represent a limitation to the interpretation of PSGL-1 expression and its role in this group of people. Indeed, due to the very limited number of newly diagnosed HIV positive patients in whom ART is not immediately initiated, we encountered considerable difficulty recruiting participants that could have increased the ART-naïve sample size. Furthermore, the absence of reliable data on the duration of HIV infection limits our ability to fully assess the impact of infection chronicity on PSGL-1 expression. In many cases, individuals are diagnosed well after seroconversion, and early HIV infection often presents with mild or nonspecific symptoms that may go unnoticed or unreported. Consequently, clinical or laboratory markers that precisely determine the timing of infection are frequently unavailable. Retrospective data collection is also constrained by incomplete medical records and participant recall bias. In our study, such information was particularly difficult to obtain from participants who had already initiated ART. Even among newly diagnosed ART-naïve individuals, the duration of infection could not be reliably documented, as patients were unable to provide this information. Similarly, the cross-sectional design of this study limits the ability to establish causal relationships between PSGL-1 expression and disease progression or immune reconstitution.

## 5. Conclusions

In summary, our observations underscore the dynamic role of PSGL-1 expression in the context of HIV infection. Indeed, PSGL-1 is markedly overexpressed in ART-treated individuals but is suppressed in their ART-naïve counterparts. Moreover, while HIV infection promotes the formation of CD4^+^ T cell–platelet aggregates in general, we observed that in both ART-treated and ART-naïve individuals, those with elevated PSGL-1 expression also exhibit higher proportions of CD4^+^ T cell–platelet aggregates. Interestingly, our observations reveal a compelling link between inflammation and microbial translocation as drivers of PSGL-1 expression, although a significant positive association between PSGL-1 expression and inflammatory and translocation markers was observed exclusively in ART-treated individuals. Furthermore, our observations reveal that the downstream signaling triggered by PSGL-1 engagement with P-selectin on platelets contributes to the shaping of CD4^+^ T cell phenotypes, fostering the expression of Caspase-3, Bcl-2, and PD-1. Overall, our observations suggest that in the context of HIV infection, inflammation, and/or microbial translocation may drive PSGL-1 expression and facilitate the formation of CD4^+^ T cell–platelet aggregates. This process may, in turn, influence CD4^+^ T cell dynamics, contributing either to their depletion, survival, or exhaustion. Further directed studies are warranted to confirm, validate, or, indeed, potentially refute our observations.

## Figures and Tables

**Table 1:** Characteristics of participants enrolled in the study.

	PLWH ART-Treated		*p*-Value	PLWH ART-Naïve		*p*-Value	HIV-Negative (n = 26)
	CD4^+^ < 200 (n = 26)	CD4^+^ ≥ 200 (n = 26)		CD4^+^ < 200 (n = 15)	CD4^+^ ≥ 200 (n = 24)		
Age (years) [Median (IQR)]	49.5 (13)	47 (14)	0.213	44 (23)	46 (15)	0.881	45 (3)
Sex			1			0.95	
Male [n(%)]	21 (80.8)	21 (80.8)		12 (80)	19 (79.16)		21 (80.8)
Female [n(%)]	5 (19.2)	5 (19.2)		3 (20)	5 (20.84)		5 (19.2)
CD4^+^ count (cells/µL) [Median (IQR)]	157 (52)	482 (303)	<0.001	141 (77)	407 (139.5)	<0.001	N/A
CD8^+^ count (cells/µL) [Median (IQR)]	458.5 (217)	790 (316)	<0.001	761 (714)	929.5 (772.5)	0.245	N/A
CD4^+^/CD8^+^ ratio [Median (IQR)]	0.25 (0.24)	0.64 (0.46)	<0.001	0.18 (0.19)	0.39 (0.22)	<0.001	N/A
Nadir CD4^+^ count (cells/µL) [Median (IQR)]	37.5 (66)	230 (201)	<0.001	N/A	N/A	-	N/A
HIV VL [Median RNA copies/mL (IQR)]	N/A	N/A	-	208,000 (306,200)	59,800 (293,550)	0.074	N/A
ART duration (years) [Median (IQR)]	5 (3)	5 (4)	0.788	N/A	N/A	-	N/A
Current ART regimens			0.004	N/A	N/A		N/A
NRTI + INSTI [n(%)]	11 (42.3%)	21 (80.8)					
NRTI + NNRTI [n(%)]	15 (57.7%)	5 (19.2)					
Duration of current ART regimens (years) [Median (IQR)]				N/A	N/A		N/A
NRTI + INSTI	3 (4)	2 (1)	0.441				
NRTI + NNRTI	5 (3)	4 (4.5)	0.355				
Number of ART regimens received [Median (IQR)]	1 (1)	2 (1)	0.328	N/A	N/A		N/A

N/A: not applicable.

## Data Availability

Upon request, and subject to review, the corresponding author will provide the data that support the findings of this study.

## References

[1] Tinoco R., Bradley L.M. (2017). Targeting the PSGL-1 pathway for immune modulation. Immunotherapy.

[2] Almulki L., Noda K., Amini R., Schering A., Garland R.C., Nakao S., Nakazawa T., Hisatomi T., Thomas K.L., Masli S. (2009). Surprising up-regulation of P-selectin glycoprotein ligand-1 (PSGL-1) in endotoxin-induced uveitis. FASEB J..

[3] Somers W.S., Tang J., Shaw G.D., Camphausen R.T. (2000). Insights into the molecular basis of leukocyte tethering and rolling revealed by structures of P- and E-selectin bound to SLe(X) and PSGL-1. Cell.

[4] Xu H., Manivannan A., Jiang H.R., Liversidge J., Sharp P.F., Forrester J.V., Crane I.J. (2004). Recruitment of IFN-gamma-producing (Th1-like) cells into the inflamed retina in vivo is preferentially regulated by P-selectin glycoprotein ligand 1:P/E-selectin interactions. J. Immunol..

[5] Schumacher A., Liebers U., John M., Gerl V., Meyer M., Witt C., Wolff G. (2005). P-selectin glycoprotein ligand-1 (PSGL-1) is up-regulated on leucocytes from patients with chronic obstructive pulmonary disease. Clin. Exp. Immunol..

[6] Borges E., Eytner R., Moll T., Steegmaier M., Campbell M.A., Ley K., Mossmann H., Vestweber D. (1997). The P-selectin glycoprotein ligand-1 is important for recruitment of neutrophils into inflamed mouse peritoneum. Blood.

[7] Asaduzzaman M., Mihaescu A., Wang Y., Sato T., Thorlacius H. (2009). P-selectin and P-selectin glycoprotein ligand 1 mediate rolling of activated CD8^+^ T cells in inflamed colonic venules. J. Investig. Med..

[8] Martín-Fontecha A., Baumjohann D., Guarda G., Reboldi A., Hons M., Lanzavecchia A., Sallusto F. (2008). CD40L^+^ CD4^+^ memory T cells migrate in a CD62P-dependent fashion into reactive lymph nodes and license dendritic cells for T cell priming. J. Exp. Med..

[9] Ley K., Kansas G.S. (2004). Selectins in T-cell recruitment to non-lymphoid tissues and sites of inflammation. Nat. Rev. Immunol..

[10] Lam F.W., Burns A.R., Smith C.W., Rumbaut R.E. (2011). Platelets enhance neutrophil transendothelial migration via P-selectin glycoprotein ligand-1. Am. J. Physiol. Heart Circ. Physiol..

[11] Martinez M., Joffraud M., Giraud S., Baïsse B., Bernimoulin M.P., Schapira M., Spertini O. (2005). Regulation of PSGL-1 Interactions with L-selectin, P-selectin, and E-selectin: Role of human fucosyltransferase-IV and -VII. J. Biol. Chem..

[12] Liu Y., Fu Y., Wang Q., Li M., Zhou Z., Dabbagh D., Fu C., Zhang H., Li S., Zhang T. (2019). Proteomic profiling of HIV-1 infection of human CD4^+^ T cells identifies PSGL-1 as an HIV restriction factor. Nat. Microbiol..

[13] Fu Y., He S., Waheed A.A., Dabbagh D., Zhou Z., Trinité B., Wang Z., Yu J., Wang D., Li F. (2020). PSGL-1 restricts HIV-1 infectivity by blocking virus particle attachment to target cells. Proc. Natl. Acad. Sci. USA.

[14] Liu Y., Song Y., Zhang S., Diao M., Huang S., Li S., Tan X. (2020). PSGL-1 inhibits HIV-1 infection by restricting actin dynamics and sequestering HIV envelope proteins. Cell Discov..

[15] Burnie J., Persaud A.T., Thaya L., Liu Q., Miao H., Grabinsky S., Norouzi V., Lusso P., Tang V.A., Guzzo C. (2022). P-selectin glycoprotein ligand-1 (PSGL-1/CD162) is incorporated into clinical HIV-1 isolates and can mediate virus capture and subsequent transfer to permissive cells. Retrovirology.

[16] Connor R., Jones L.D., Qiu X., Thakar J., Maggirwar S.B. (2017). Frontline Science: c-Myc regulates P-selectin glycoprotein ligand-1 expression in monocytes during HIV-1 infection. J. Leukoc. Biol..

[17] Liang H., Duan Z., Li D., Li D., Wang Z., Ren L., Shen T., Shao Y. (2015). Higher levels of circulating monocyte-platelet aggregates are correlated with viremia and increased sCD163 levels in HIV-1 infection. Cell Mol. Immunol..

[18] Nakanjako D., Kiragga A.N., Musick B.S., Yiannoutsos C.T., Wools-Kaloustian K., Diero L., Oyaro P., Lugina E., Ssali J.C., Kambugu A. (2016). Frequency and impact of suboptimal immune recovery on first-line antiretroviral therapy within the International Epidemiologic Databases to Evaluate AIDS in East Africa. AIDS.

[19] Tinoco R., Neubert E.N., Stairiker C.J., Henriquez M.L., Bradley L.M. (2021). PSGL-1 Is a T Cell Intrinsic Inhibitor That Regulates Effector and Memory Differentiation and Responses During Viral Infection. Front. Immunol..

[20] Dillon S.M., Lee E.J., Kotter C.V., Austin G.L., Dong Z., Hecht D.K., Gianella S., Siewe B., Smith D.M., Landay A.L. (2014). An altered intestinal mucosal microbiome in HIV-1 infection is associated with mucosal and systemic immune activation and endotoxemia. Mucosal Immunol..

[21] DeRogatis J.M., Viramontes K.M., Neubert E.N., Tinoco R. (2021). PSGL-1 Immune Checkpoint Inhibition for CD4^+^ T Cell Cancer Immunotherapy. Front. Immunol..

[22] Babu H., Ambikan A.T., Gabriel E.E., Svensson Akusjärvi S., Palaniappan A.N., Sundaraj V., Mupanni N.R., Sperk M., Cheedarla N., Sridhar R. (2019). Systemic Inflammation and the Increased Risk of Inflamm-Aging and Age-Associated Diseases in People Living With HIV on Long Term Suppressive Antiretroviral Therapy. Front. Immunol..

[23] Hileman C.O., Funderburg N.T. (2017). Inflammation, Immune Activation, and Antiretroviral Therapy in HIV. Curr. HIV/AIDS Rep..

[24] Dai X.P., Wu F.Y., Cui C., Liao X.J., Jiao Y.M., Zhang C., Song J.W., Fan X., Zhang J.Y., He Q. (2021). Increased Platelet-CD4^+^ T Cell Aggregates Are Correlated With HIV-1 Permissiveness and CD4^+^ T Cell Loss. Front. Immunol..

[25] Green S.A., Smith M., Hasley R.B., Stephany D., Harned A., Nagashima K., Abdullah S., Pittaluga S., Imamichi T., Qin J. (2015). Activated platelet-T-cell conjugates in peripheral blood of patients with HIV infection: Coupling coagulation/inflammation and T cells. AIDS.

[26] Zaongo S.D., Song Y., Chen Y. (2025). P-selectin glycoprotein ligand-1 and cardiovascular diseases: From a general perspective to an HIV infection context. Front. Cardiovasc. Med..

[27] Haddad W., Cooper C.J., Zhang Z., Brown J.B., Zhu Y., Issekutz A., Fuss I., Lee H.O., Kansas G.S., Barrett T.A. (2003). P-selectin and P-selectin glycoprotein ligand 1 are major determinants for Th1 cell recruitment to nonlymphoid effector sites in the intestinal lamina propria. J. Exp. Med..

[28] Zaongo S.D., Chen Y. (2023). PSGL-1, a Strategic Biomarker for Pathological Conditions in HIV Infection: A Hypothesis Review. Viruses.

[29] Lévesque J.P., Zannettino A.C., Pudney M., Niutta S., Haylock D.N., Snapp K.R., Kansas G.S., Berndt M.C., Simmons P.J. (1999). PSGL-1-mediated adhesion of human hematopoietic progenitors to P-selectin results in suppression of hematopoiesis. Immunity.

[30] Hope J.L., Otero D.C., Bae E.A., Stairiker C.J., Palete A.B., Faso H.A., Lin M., Henriquez M.L., Roy S., Seo H. (2023). PSGL-1 attenuates early TCR signaling to suppress CD8^+^ T cell progenitor differentiation and elicit terminal CD8^+^ T cell exhaustion. Cell Rep..

[31] Chen S.C., Huang C.C., Chien C.L., Jeng C.J., Su H.T., Chiang E., Liu M.R., Wu C.H., Chang C.N., Lin R.H. (2004). Cross-linking of P-selectin glycoprotein ligand-1 induces death of activated T cells. Blood.

[32] Susin S.A., Zamzami N., Castedo M., Hirsch T., Marchetti P., Macho A., Daugas E., Geuskens M., Kroemer G. (1996). Bcl-2 inhibits the mitochondrial release of an apoptogenic protease. J. Exp. Med..

[33] Yang W.H., Nussbaum C., Grewal P.K., Marth J.D., Sperandio M. (2012). Coordinated roles of ST3Gal-VI and ST3Gal-IV sialyltransferases in the synthesis of selectin ligands. Blood.

